# Comprehensive single-shot biophysical cytometry using simultaneous quantitative phase imaging and Brillouin spectroscopy

**DOI:** 10.1038/s41598-022-23049-4

**Published:** 2022-10-31

**Authors:** Zachary A. Steelman, Zachary N. Coker, Anna Sedelnikova, Mark A. Keppler, Allen S. Kiester, Maria A. Troyanova-Wood, Bennett L. Ibey, Joel N. Bixler

**Affiliations:** 1 Air Force Research Laboratory, JBSA Fort Sam Houston, San Antonio, TX 78234 USA; 2 SAIC, San Antonio, TX USA; 3 Oak Ridge Institute for Science and Education, Oak Ridge, TN USA

**Keywords:** Imaging and sensing, Microscopy, Biophysics

## Abstract

Single-cell analysis, or cytometry, is a ubiquitous tool in the biomedical sciences. Whereas most cytometers use fluorescent probes to ascertain the presence or absence of targeted molecules, biophysical parameters such as the cell density, refractive index, and viscosity are difficult to obtain. In this work, we combine two complementary techniques—quantitative phase imaging and Brillouin spectroscopy—into a label-free image cytometry platform capable of measuring more than a dozen biophysical properties of individual cells simultaneously. Using a geometric simplification linked to freshly plated cells, we can acquire the cellular diameter, volume, refractive index, mass density, non-aqueous mass, fluid volume, dry volume, the fractional water content of cells, both by mass and by volume, the Brillouin shift, Brillouin linewidth, longitudinal modulus, longitudinal viscosity, the loss modulus, and the loss tangent, all from a single acquisition, and with no assumptions of underlying parameters. Our methods are validated across three cell populations, including a control population of CHO-K1 cells, cells exposed to tubulin-disrupting nocodazole, and cells under hypoosmotic shock. Our system will unlock new avenues of research in biophysics, cell biology, and medicine.

## Introduction

Cytometry—the assignment of quantifiable metrics to individual cells, and their subsequent analysis—is omnipresent in the biomedical sciences. Basic research and medical diagnostics rely heavily on the ability to numerically assess the functional unit of life^1–3^.

While remarkable advances have been made in recent years^4^, most cytometric devices have relied on two traditional imaging techniques: fluorescence labelling and elastic scattering^5^. The former utilizes targeted fluorophores to report on the presence of individual molecules, while the latter gives rudimentary information regarding cellular morphology from the angular distribution of elastically scattered photons.

Techniques within this paradigm have proven remarkably effective, however, they generally offer information regarding a finite, predetermined list of molecular targets^6^ or give only rough approximations of the cellular morphology^7^. Herculean efforts have been made to engineer ingenious molecular and atomic probes^8,9^, yet the paradigm of searching amongst a small, predetermined set of individual molecules persists.

Meanwhile, the rise of cellular biomechanics^10^, along with computational biophysics and associated multiphysics tools^11^ requires detailed descriptions of the physical, rather than molecular, state of the cell^12^. Biophysical parameters such as the refractive index (RI), mass density, mechanical modulus, viscosity, and water content are not easily measured using traditional cytometry, and greatly inform how a cell will respond to mechanical, optical, electromagnetic, and acoustic insults. These parameters in turn have numerous applications in fields such as tumorigenesis^13^, cellular differentiation^14^, and cancer therapeutics^15^. The rise of image-based single-cell sorting^16^ and single-cell sequencing^17^ further suggests the utility in modernizing cytometry technologies, by presenting an opportunity to fill the knowledge gap between macromolecular expression and biophysical phenotype^18^.

This work combines two advanced optical techniques—quantitative phase imaging (QPI) and Brillouin spectroscopy—into a single image cytometry platform which computes more than a dozen biophysical parameters, label-free, from a single snapshot acquisition. Quantitative phase imaging is an interferometric approach to microscopy which uses the phase delay of light as a label-free contrast mechanism^19^, while Brillouin spectroscopy uses inelastically scattered photons to probe the viscoelastic properties of biological systems^20,21^. We implement synchronous and co-localized acquisition of a QPI hologram and Brillouin spectrum of a cell to obtain complementary information, such that several biophysical parameters are measured which are not obtainable using either system individually. Geometric measurements of the cellular morphology are leveraged alongside a two-component mixture model to obtain the cellular refractive index, density, and water content from a single phase image.

**Figure 1:**
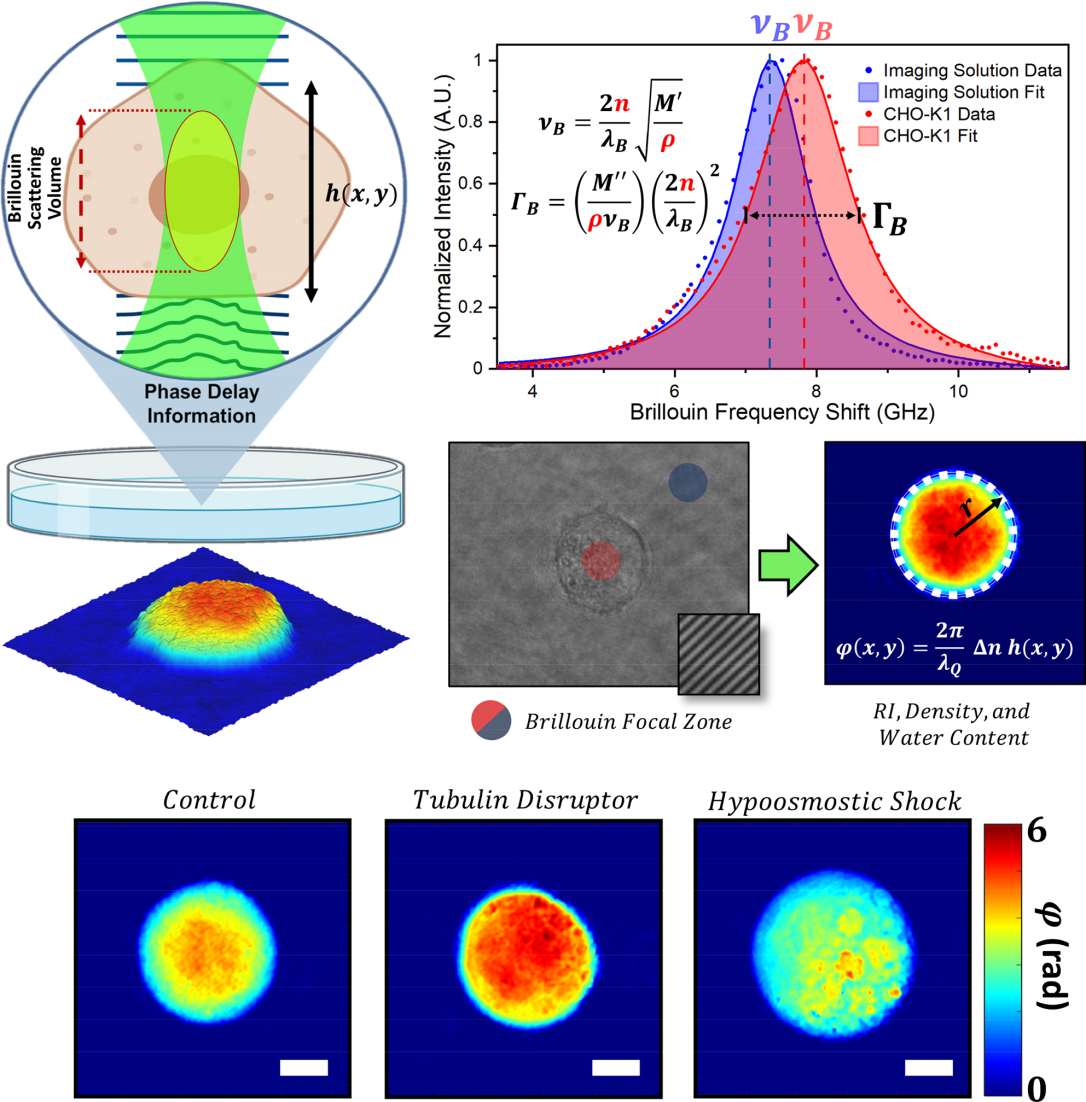
Conceptual layout of the proposed system. Phase imaging of cells with a known geometry provides their refractive index and density, which are necessary inputs to numerous Brillouin parameters, while also computing the water content by mass and volume. From this information, several viscoelastic properties are directly computable, without assumptions of the underlying parameters. Three representative phase images are included for each exposure condition. Scale bars 5 µm. Diagram created in part with BioRender (https://biorender.com/).

A conceptual diagram of our methods is shown in Fig. 1. Recent work has shown that known geometries may decouple the sample thickness and refractive index ambiguity in QPI^22^. In this work, we take advantage of the innate spherical geometry of cells prior to adhesion, by imaging shortly after seeding cells on a glass-bottom Petri dish^23^. Knowledge of the cell’s geometry allows us to extract the averaged refractive index for each cell ($\overline{{n }_{cell}}$) from the QPI hologram. Geometric considerations, along with the cellular dry mass (${m}_{dry}$^24^) or the mass of non-aqueous constituent macromolecules, are utilized to compute the cell’s mass density ($\rho$), fluid volume (${V}_{fluid}$), mass fraction of water (${u}_{{H}_{2}O}$) and volume fraction of water (${\theta }_{{H}_{2}O}$). A green laser, centered within the QPI field of view, is used to detect the Brillouin shift (${\upsilon }_{B}$) and Brillouin linewidth (${\Gamma }_{B}$), which are linked to mechanical properties through functions involving the mass density and refractive index^25^. Rather than assuming these parameters^26–28^, we utilize the mass density and refractive index obtained from QPI to directly compute the longitudinal modulus ($M{^{{\prime}}}$), longitudinal viscosity ($\eta$), the loss modulus ($M{^{{\prime\prime}}}$), and the loss tangent ($\mathrm{tan}\varphi$).

In total, we report 15 parameters which describe the biophysical and biomechanical state of the cell from a single, co-localized, snapshot acquisition. We note here that very recently, QPI (or optical diffraction tomography, a three-dimensional analogue of QPI which constructs refractive index tomograms from dozens or hundreds of QPI images^29^) have been utilized in parallel with Brillouin microscopy to investigate some of the parameters listed here. These studies are noteworthy and impressive, however, they involved sequential, rather than simultaneous acquisition of Brillouin and QPI or optical diffraction tomography data, either from distinct optical systems^30,31^, or sequentially using moveable mirrors^32^. Perhaps most importantly, our technique permits direct measurement of the intracellular water content (a major topic of interest in Brillouin imaging) from a single QPI hologram. This is a significant benefit of our measurement scheme. Among our diverse measurements, we present results comparing the Brillouin shift and intracellular water content across three cell populations, providing direct measurement of the much discussed and controversial relationship between these parameters^33,34^. Lastly, our system is optimized for cytometry rather than sub-cellular imaging, as averaged values for the refractive index and density are reported for each cell. This significantly reduces the acquisition time compared with techniques using optical diffraction tomography, in which > 100 QPI holograms are generally required across various illumination angles.

To validate our device, CHO-K1 cells in three sub-populations were interrogated by our system. These include a control population, a population of cells exposed to a microtubule disrupting agent, and cells under hypoosmotic shock. For each condition, 15 biophysical parameters were reported, and correlations between parameters were observed both within and across sub-populations. We hope this system will lead to new insights in biophysical analysis, as well as provide new methods to researchers working in both phase imaging and Brillouin microscopy.

## Methods

### Instrumentation

**Figure 2:**
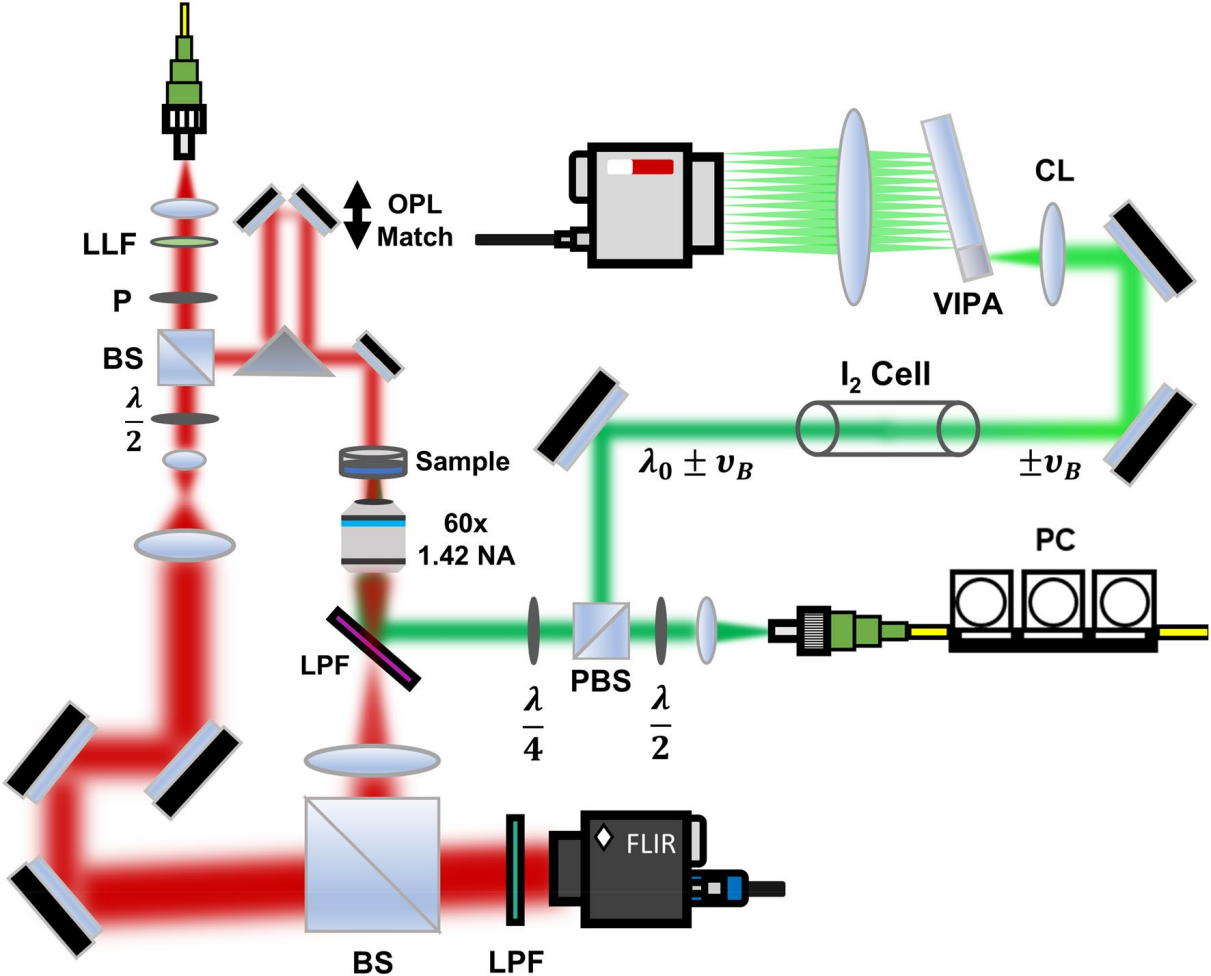
Dual QPI and Brillouin spectroscopy platform for parallel computing of biophysical parameters of single cells. Both cameras are simultaneously triggered to ensure concurrent acquisition of the QPI interferogram and Brillouin spectrum. *LLF* laser line filter, *P* polarizer, *LPF* long pass filter, *CL* cylindrical lens, *PC* polarization controller, *VIPA* virtually imaged phased array, *PBS* polarizing beamsplitter, *BS* non-polarizing beamsplitter.

The combined system architecture is displayed in Fig. 2. An off-axis quantitative phase microscope was built according to previously reported protocols^24,35^. Light from a supercontinuum laser (NKT Fianium) was first filtered using an acousto-optic tunable filter (AOTF) module to a center wavelength of 632.8 nm, with a bandwidth of approximately 3.8 nm. The light was coupled to a single-mode fiber to restrict the illumination to a single spatial mode, at which point the light was further filtered using a laser line filter (LLF) with a bandwidth of ~ 1 nm (ThorLabs, transmission > 50%) and linearly polarized. The beam was split using a 90:10 non-polarizing beamsplitter cube into sample and reference pathways. In the sample path, a delay arm was added using a prism and two silvered mirrors mounted on a translation stage. This provided the means to match the optical pathlength between arms. The sample beam was collected using an objective lens (UPLXAPO 60XO, 60×, NA = 1.42, Olympus Corp) and imaged onto the detector using a tube lens (f = 180 mm achromat, 2″ diameter). In the reference path, a 4*f* telescope was used to magnify the beam to encompass the surface of the sensor (FLIR Blackfly S, Sony IMX250 sensor, 3.45 µm pixels), while a $\frac{\lambda }{2}$ waveplate and neutral density filter were used to match the polarization and intensity of the sample arm, respectively, to improve fringe visibility. A 2″ non-polarizing beamsplitter cube was used to combine the two beams at a slight angle ($\theta$ ~ 5°), allowing for separation of the sample contribution by spatial filtering in the frequency domain^36^. The integration time for the QPI detector was 15 ms, chosen to fill the well depth of the camera without risk of saturation. The beam power incident on the sample for the QPI modality was approximately 70 µW, spread across a collimated Gaussian beam diameter of approximately 1 mm.

The Brillouin microscopy arm of the system was based on a design described in previous publications^37,38^. A tunable, ultra-narrow (< 1 kHz) single-frequency laser (NKT Koheras ADJUSTIK/BOOSTIK Y10) with 1064 nm center wavelength and a second harmonic generation crystal were used to produce a 532 nm output beam, which was fiber-coupled to produce a clean Gaussian spatial mode for easy coupling into the QPI system. Polarization paddles and a half-wave plate were used to optimize throughput through a polarizing beamsplitter, after which a quarter-wave plate was used to induce circular polarization prior to the sample. The Brillouin beam power incident on the sample was approximately 3.8 mW. The quarter-wave plate and polarizing beamsplitter ensure that Brillouin-scattered photons are efficiently coupled back into the spectrometer.

A 532 nm long pass filter, or LPF (RazorEdge LPD02-532RU, Semrock) was inserted between the objective and tube lenses in the QPI system to allow easy insertion of the Brillouin beam. Collimated light from the Brillouin laser was deflected by the long pass filter to the same microscope objective used for QPI imaging. Light was collected from the sample in a 180° backscattered configuration by the same objective, and Brillouin scattered photons (${\lambda }_{B}$ ~ 532 nm) were reflected by the long pass filter, without distorting the QPI image (${\lambda }_{Q}$ ~ 633 nm). Brillouin signal was passed through an ultra-narrow I_2_ gas absorption-based notch filter (GC19100-I, ThorLabs, Inc.) to reduce elastically scattered photons. Prior to the experiment, our laser was tuned to an iodine absorption line, as confirmed via suppression of the elastic peak. While this was sufficient for our purposes, manual retuning of the laser was occasionally required, and a frequency locking scheme is preferable for future systems. From here, the Brillouin beam was passed to a virtually imaged phased array (VIPA)-based Brillouin spectrometer, consisting of a cylindrical lens, VIPA, and spherical lens to efficiently separate the Brillouin-scattered photons from the elastic scattering. Output from the VIPA was collected by a CMOS camera (ORCA-Flash4.0 C13440-20CU, Hamamatsu Photonics K.K.). Our VIPA had a free spectral range of 29.95 GHz (OP-6721-3371-2, Light Machinery, Inc.), while our Brillouin spectrometer exhibited a spectral contrast of − 60 dB and spectral resolution of $\delta \nu$ = 398 ± 18 MHz, as measured by the spectral width of our laser. The repeatability of Brillouin shifts, determined by the standard deviation of repeated measurements of the same cell over ~ 1 min, was consistently ≤ 10 MHz.

Synchronous temporal acquisition between the QPI and Brillouin cameras was coordinated using a National Instruments DAQ card (PCIe-6612). Custom LabVIEW software was designed for live visualization of the QPI interferogram, and to ensure that the cell was in focus. Temporally coincident with the QPI acquisition, a TTL signal was sent to the Hamamatsu camera to trigger the Brillouin acquisition. The integration time was 15 ms for the QPI camera, and 2 s for the Brillouin camera, to ensure a sufficient signal-to-noise ratio in the recorded spectrum.

In addition to synchronizing the temporal acquisitions, it is important that the focal volume of the Brillouin beam be centered on the cell of interest, both axially and laterally. To find the lateral location of the Brillouin spot, an additional 550 nm long pass filter, placed in front of the QPI camera to filter any residual light from the Brillouin beam, was temporarily removed. This allowed a tiny portion of elastically scattered light from the Brillouin focal zone to be imaged onto the QPI camera. This spot was marked with a crosshair on LabVIEW’s live rendering of the interferogram to indicate the spatial location of the Brillouin interrogation zone. The axial location of the beam was found by mechanically translating a glass-bottomed dish along the beam axis until a clear reflection from the glass-water interface was observed as increased elastic scattering at the Brillouin camera. From this point, a graduated micrometer was used to translate the dish such that the Brillouin focal zone was localized approximately six microns above the glass–water interface, or approximately half the diameter of the CHO-K1 cells used in this study. Slight defocus in the QPI image was corrected by translating the QPI camera to the proper image plane, and the co-localization of the Brillouin and QPI modalities was verified by an independent experiment showing a clear distinction (*p* < 10^–5^) between measurements of the Brillouin shift in a population of cells (7.863 ± 0.049 GHz) and cell media (7.308 ± 0.037 GHz) whose locations were identified using QPI.

### Data processing

#### QPI

QPI interferograms were processed according to standard protocols. A two-dimensional fast Fourier transform was applied to each raw interferogram to isolate the interferometric term of interest. From here, this term was cropped, recentered, and inverse-transformed to produce the complex field. The argument of the complex field was taken to isolate the phase contribution, and a two-dimensional phase unwrapping algorithm based on the transport of intensity equation was applied to remove any 2π-ambiguities^39^. Finally, the background phase trend was removed by manually segmenting the cells, and then fitting a two-dimensional polynomial to the remaining background regions. While manual cropping of the cells was not particularly time-consuming for the sample sizes in this work, automatic segmentation schemes using a trained neural network are being developed to fully automate the QPI data processing pipeline. Further details on QPI image processing computations may be found in the literature^24,35^.

#### Brillouin spectroscopy

Spectral analysis was performed using a custom Python script (Python 3.8.5). Brillouin peaks were individually located and fit to a Lorentzian function using a least-squares fitting protocol. The pixel position of each Brillouin peak was then converted to a frequency shift in gigahertz using a polynomial interpolation based on the VIPA’s 29.95 GHz free spectral range. The Brillouin frequency shift was then determined as half the distance between the respective Stokes and anti-Stokes peak centers, and the Brillouin linewidth was defined by the FWHM of the Lorentzian fit of each respective peak, corrected for instrumental broadening. The average signal-to-noise ratio (SNR) for Brillouin measurements of cells across all samples was approximately 32 dB, calculated using previously reported methods^40^.

### Parameter computation

The refractive index, density, longitudinal modulus, and other biophysical parameters of each cell were computed as follows. From each phase image, $\varphi \left(x,y\right)$, the mean refractive index of the cell was computed utilizing the assumed spherical geometry, according to previously reported methods^22,23,41^. Briefly, a projected thickness map $h\left(x,y\right)$ was generated for each cell by applying a circular Hough transform to the phase profile of the cell. By determining the location and diameter of the (freshly plated) spherical cell, its projected thickness is easily computed via geometric considerations. From calibration experiments with polystyrene microsphere size standards (N = 10 images each of 10 µm diameter microspheres, standard deviation 0.85 µm, immersed in Cargille refractive index liquids, RI = 1.56, 1.57, and 1.58, for a total of 30 images), we found that this method consistently overestimated the diameter of the spheres by ~ 8%, likely due to Gaussian blurring caused by the objective lens’s point spread function. This factor was used to adjust the diameter resulting from the Hough transform to correctly identify the diameter $D$ of the spherical cell, with fitting accuracy confirmed by visual inspection.

From this measurement, a refractive index map was computed using

1 $$\Delta n\left( {x,y} \right) = \frac{{\lambda _{Q} }}{{2\pi }}\frac{{\varphi \left( {x,y} \right)}}{{h\left( {x,y} \right)}},$$

where ${\lambda }_{Q}$ is the QPI illumination wavelength. The average refractive index (RI) of the cell $\overline{{n }_{cell}}$ was computed using the average of points within the cell boundary (excluding points within a tenth of the cell’s radius from the cell boundary to avoid edge effects) and adjusting for the refractive index of the imaging medium, ${n}_{medium}$ ~ 1.335, as measured using a commercial refractometer (Fisherbrand™, HDR-P6, accuracy ± 0.0003):

2 $$\overline{{n }_{cell}}= \frac{1}{N}\sum_{\begin{array}{c}Cell\\ Area\end{array}}\Delta n\left(x,y\right)+ {n}_{medium},$$

where $N$ is the number of sample points. This produces an average RI measurement for the whole cell.

From the Brillouin spectrum, a Brillouin shift for the whole cell was measured using our Brillouin spectrometer, which satisfies the typical relation,

3 $$\nu _{B} = \frac{{2\overline{{n_{{cell}} }} }}{{\lambda _{B} }}{\text{ }}\sqrt {\frac{{M^{\prime}}}{\rho }} ,$$

where $M{^{{\prime}}}$ is the longitudinal modulus, ${\lambda }_{B}$ is the Brillouin excitation wavelength, and $\rho$ is the mass density of the cell. From this equation, the longitudinal modulus may be expressed as

4 $${M}^{^{\prime}}= \rho {\left(\frac{{{\nu }_{B}\lambda }_{B}}{2\overline{{n }_{cell}}}\right)}^{2},$$

where $\overline{{n }_{cell}}$ was obtained from the QPI image. The density $\rho$ is obtained as follows. First, we assume the cell is a two-component mixture containing both dry and fluid components. The dry mass of non-aqueous constituents^42,43^ is given by

5 $${m}_{dry}= \frac{\lambda }{2\pi \alpha }\iint \varphi \left(x,y\right)dxdy,$$

where $\alpha$ is the refractive index increment of intracellular proteins, approximately 0.2 mL/g^44,45^.

The absolute density within the cell is given by the sum of the masses of dry and fluid constituents, divided by the volume of the cell

6 $$\rho = \frac{{m}_{dry}+{m}_{fluid}}{{V}_{cell}}.$$

Using the diameter of the cell $D$, already computed from the Hough transform, yields the cell volume ${V}_{cell}$ given by

7 $${V}_{cell}= \frac{4}{3}\pi {\left(\frac{D}{2}\right)}^{3}.$$

The dry mass ${m}_{dry}$ can be used to compute a dry volume ${V}_{dry}= \frac{{m}_{dry}}{{\rho }_{dry}}$, using the average mass density of proteins ${\rho }_{dry}$ = 1.37 g/mL = 1.37 pg/μm^3^ obtained from the literature^30,46^. The fluid volume is then easily obtained as the remaining fraction of the total volume ${V}_{fluid}={V}_{cell}- {V}_{dry}$, and the fluid mass expressed as

8 $${m}_{fluid}= {\rho }_{fluid}*{V}_{fluid}={\rho }_{fluid}\left(\frac{4}{3}\pi {\left(\frac{D}{2}\right)}^{3}- \frac{{m}_{dry}}{{\rho }_{dry}}\right),$$

where ${\rho }_{fluid}$ is assumed to be close to that of water, ~ 1 g/mL. Thus, the absolute density within the cell is given by

9 $$\rho = \frac{{m}_{dry}+{\rho }_{fluid}\left(\frac{4}{3}\pi {\left(\frac{D}{2}\right)}^{3}- \frac{{m}_{dry}}{{\rho }_{dry}}\right)}{\frac{4}{3}\pi {\left(\frac{D}{2}\right)}^{3}}$$

and combining with Eq. (4), the longitudinal modulus may be expressed as

10 $${M}^{^{\prime}}= \left(\frac{{m}_{dry}+{\rho }_{fluid}\left(\frac{4}{3}\pi {\left(\frac{D}{2}\right)}^{3}- \frac{{m}_{dry}}{{\rho }_{dry}}\right)}{\frac{4}{3}\pi {\left(\frac{D}{2}\right)}^{3}}\right){\left(\frac{{{\nu }_{B}\lambda }_{B}}{2\overline{{n }_{cell}}}\right)}^{2}.$$

Note that the spherical geometry of the cell allows us to explicitly compute the volume of the cell as well as both dry and fluid density contributions. Finally, the volumetric water content (${\theta }_{{H}_{2}O}$) of the cell is easily obtained by dividing the fluid volume by the cell volume, ${\theta }_{{H}_{2}O}= {V}_{fluid}/{V}_{cell}$, while the gravimetric (by mass) water content is equivalently obtained by dividing the fluid mass by the sum of the fluid and dry masses, ${u}_{{H}_{2}O}=\frac{{m}_{fluid}}{{m}_{fluid}+{{m}_{dry}}}$. Both quantities are expressed as percentages for convenience.

From the Brillouin linewidth ${\Gamma }_{B}$, Brillouin shift ${\nu }_{B}$, and refractive index and density obtained from QPI, the loss modulus ${M}^{{^{{\prime\prime}}}}$ of cells may be computed according to the relation^25,47,48^

11 $${M}^{{^{{\prime\prime}}}}=\rho {\Gamma }_{B}{\nu }_{B}{\left(\frac{{\lambda }_{B}}{2\overline{{n }_{cell}}}\right)}^{2},$$

yielding the complex modulus $M={M}^{^{\prime}}+iM{^{{\prime\prime}}}$. Using this value, the longitudinal viscosity η may be computed in units of pascal-seconds (Pa s) as previously shown^49^, where

12 $$\eta =\frac{{M}^{{^{{\prime\prime}}}}}{2\pi {\nu }_{B}}.$$

Finally, the loss tangent

13 $${\text{tan}}\varphi = \frac{{M^{\prime\prime}}}{{M^{\prime}}}$$

may be calculated^48–50^, demonstrating the relative ratio of viscous to elastic effects.

### Cell culture and experimental design

The adherent cell line CHO-K1 (ATCC^®^ CCL-61™, Chinese hamster ovary) was used for all experimental procedures. This cell line was selected because of its spherical shape for up to two hours after plating, which is necessary for our analysis protocols. Cells were cultured according to methods established by the provider. Briefly, cells were propagated in Kaighn's Modification of Ham's F-12 Medium, supplemented with 10% fetal bovine serum, 2 mM l-glutamine, and 1% volume 100 U/mL penicillin/streptomycin and maintained at 37 °C with 5% CO_2_ in air with relative humidity of 95%. Approximately 150,000 cells each were plated in No. 1.5 glass-bottomed 35 mm petri dishes (P35GC-1.5-10-C; Mattek Corp.) and then incubated in complete growth media for 30 min before imaging to allow for settling without permitting adhesion, and to promote recovery. Cells were rinsed and subsequently imaged in physiological imaging buffer solution (A14291DJ Live Cell Imaging Solution; Invitrogen). All images and measurements were taken within 90 min of plating to ensure cell sphericity.

Simultaneous QPI images and Brillouin spectra were acquired from CHO-K1 cells under three exposure conditions: a control experiment with untreated cells in physiological buffer solution, cells exposed to the chemotherapy drug nocodazole, which is known to disrupt microtubule networks, and cells exposed to a hypotonic solution made by mixing 70:30% by volume distilled water to physiological buffer solution. While nocodazole (Cat. # M1404-2MG; Sigma Aldrich) is known to alter cellular morphology, this manifests in practice as an increase in cell rounding^51^, maintaining our assumption of a spherical sample. Nocodazole was mixed with buffer solution to create a 5 µM working stock before experiments. Approximately 30 min prior to imaging, 1 mL of nocodazole stock solution was added to 1 mL of imaging buffer already in the petri dish to achieve a final 2.5 µM exposure concentration.

### Calibration of Brillouin measurements

In this work, a high numerical aperture objective (Olympus, 60 × oil immersion, NA 1.42) was used to ensure high resolution QPI images, which improves geometric characterization of the cell boundaries. Unfortunately, the use of a high-NA collection geometry can modestly alter the measurement of the Brillouin shift (${\nu }_{B}$) and the Brillouin linewidth (${\Gamma }_{B}$). To reduce the impact of this error, we intentionally underfilled the rear aperture of the objective, placing a 3 mm Brillouin beam into the center of the objective’s 11 mm stop, substantially reducing the effective numerical aperture for the Brillouin (but not QPI) component of the system. This allows simultaneously high-resolution QPI images while reducing the impact of spectral broadening in the Brillouin beam, and has the added benefit of enlarging the Brillouin focal zone to sample more of the cellular volume. We characterized the error imposed by our choice of collection geometry by taking Brillouin shift and linewidth measurements of a 1 M sucrose control solution, chosen because its Brillouin shift and linewidth are closer to that of intracellular material than deionized water. The Brillouin shift and linewidth of this solution were measured using both a low-NA objective (Olympus 10×, NA 0.3) and our test objective with its aperture underfilled by the Brillouin beam. We found a slight reduction in the Brillouin shift (from 8.407 ± 0.004 to 8.172 ± 0.005 GHz, N = 20 spectra each) using the high-NA objective with underfilled aperture in comparison with the low-NA scheme, but no perceptible change in the Brillouin linewidth (from 1.048 ± 0.010 to 1.045 ± 0.012 GHz), changes of − 2.8% and − 0.3%, respectively. These results collectively suggest that spectral broadening is a small concern in our system, which may be due in part to our choice of a 180° backscattering geometry, which is significantly less prone to these issues^52^. Brillouin shift values are reported as measured, while Brillouin linewidths are corrected for instrumental broadening by subtracting the linewidth of the instrument response function (here, 0.398 GHz, as measured using the FWHM of the unsaturated elastic peak) as previously reported^40^.

## Results

**Figure 3:**
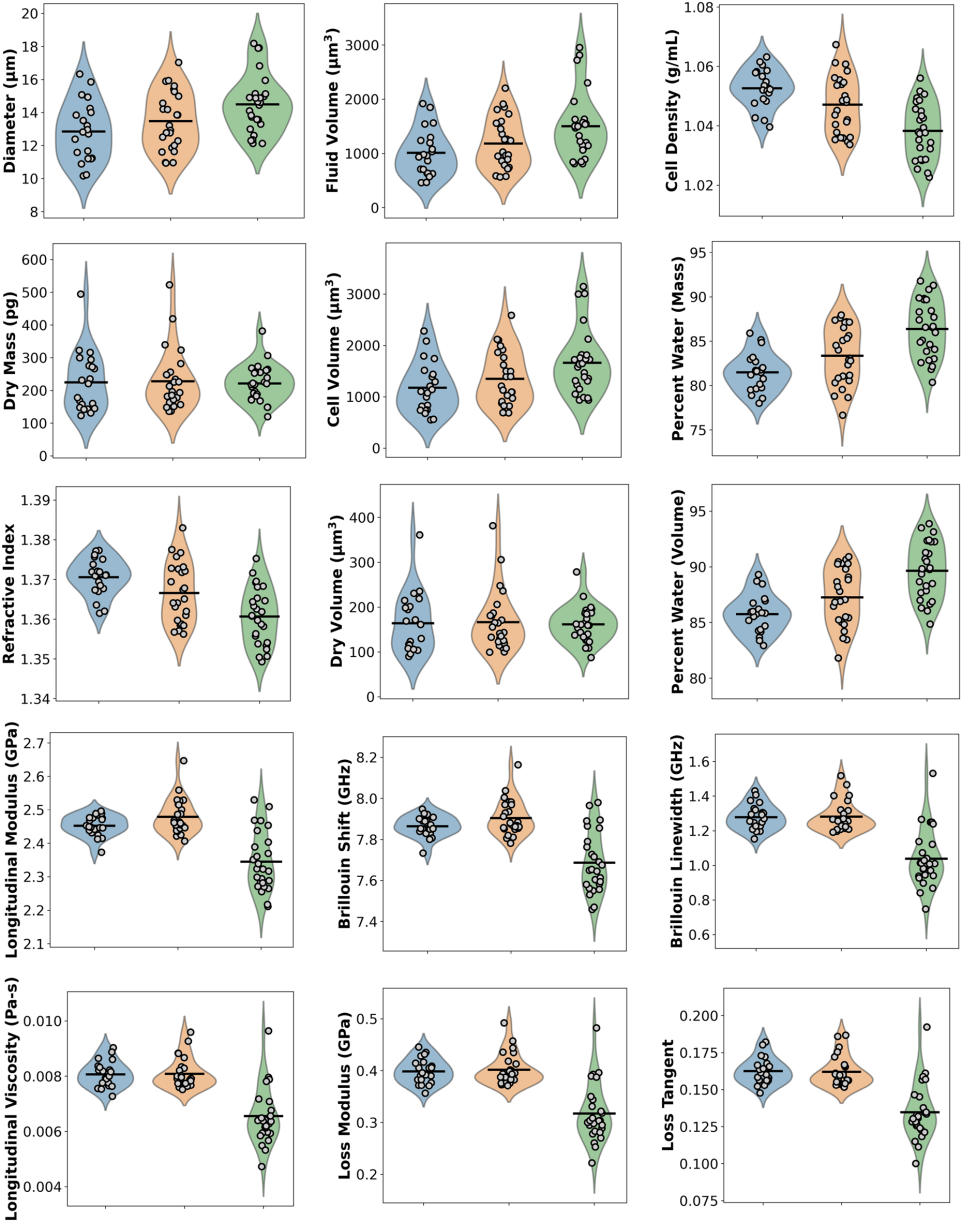
Violin plots displaying the raw data and density estimations for the 15 parameters described by our system. Blue, orange, and green represent the control, nocodazole-treated, and hypoosmotic shock conditions, respectively. Black lines indicate the population means.

**Figure 4:**
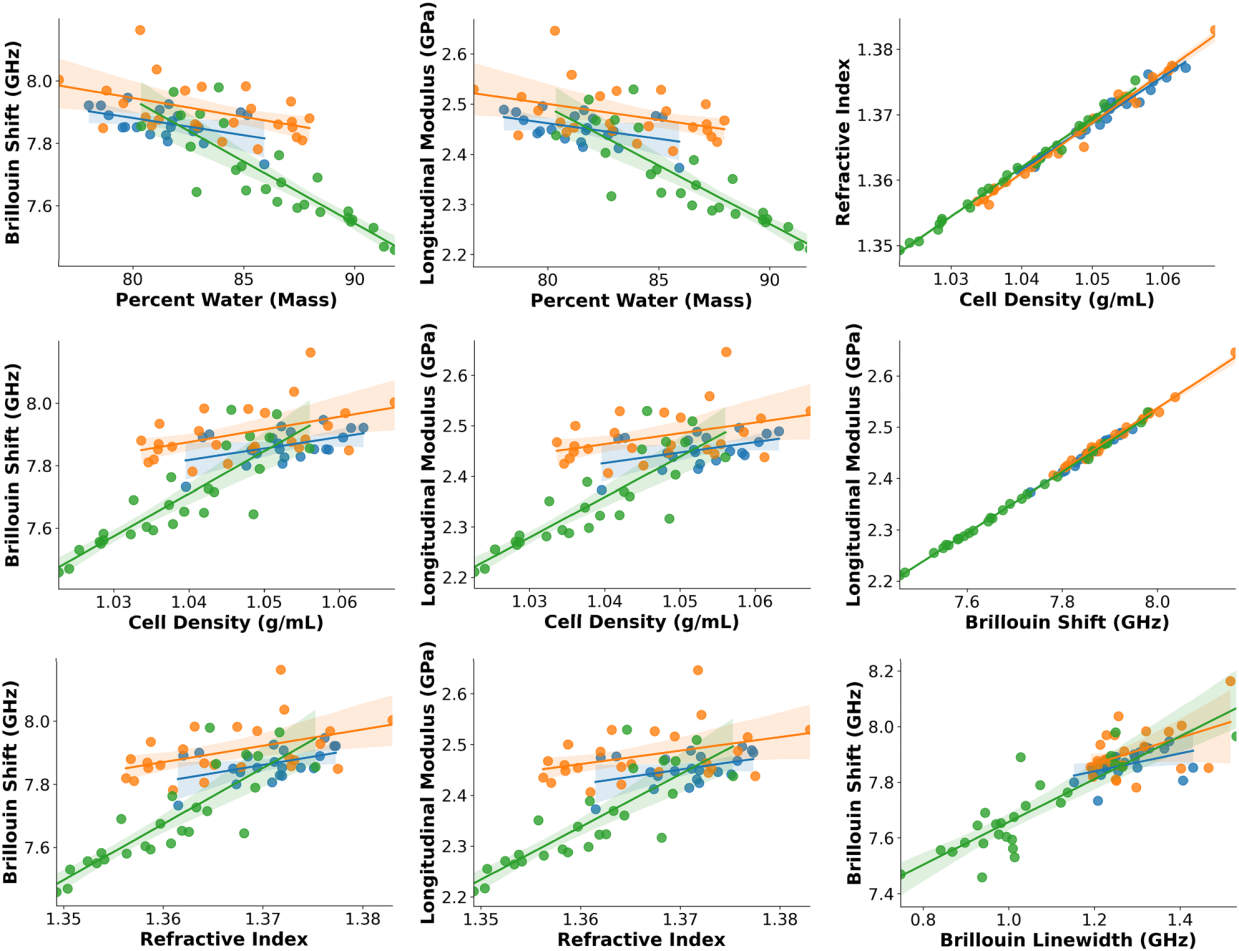
Selected correlations between parameters, with a focus on elastic properties. Best fit lines are given for each sub-population. Blue, orange, and green points represent control, nocodazole, and hypoosmotic cells, respectively. Shaded regions represent 95% confidence interval for the best fit lines.

**Figure 5:**
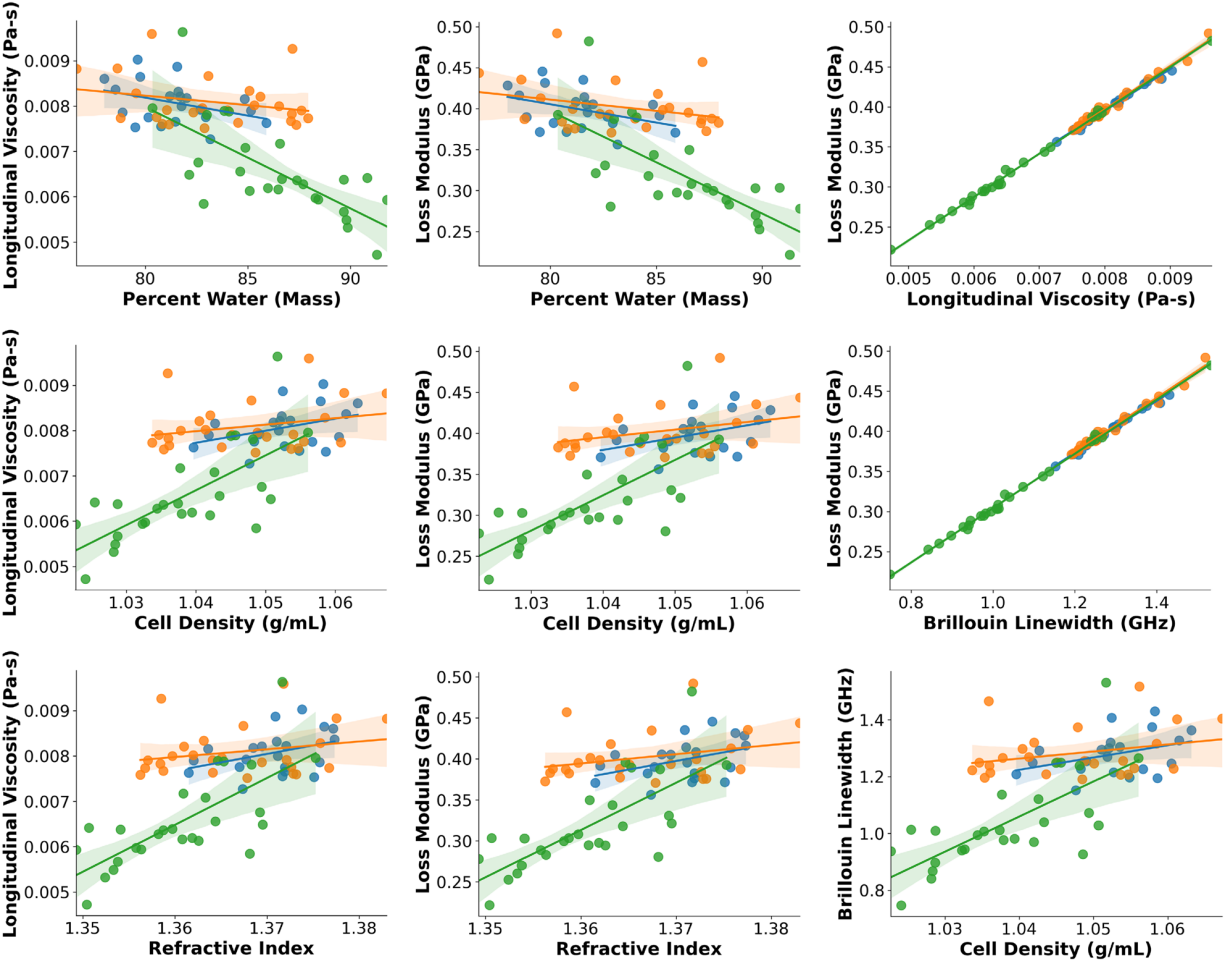
Selected correlations between parameters, with a focus on viscous properties. Best fit lines are given for each sub-population. Blue, orange, and green points represent control, nocodazole, and hypoosmotic cells, respectively. Shaded regions represent 95% confidence interval for the best fit lines.

The results from our system are demonstrated in Table 1, and visualized in Figs. 3, 4 and 5. Across the three exposure conditions, each of the biophysical properties of cells were examined across a population of N = 21, 25, and 26 cells for the control, tubulin disrupter, and hypoosmotic shock conditions, respectively. All imaging was completed within 90 min of cell plating to ensure a highly spherical geometry. All errors are reported as standard deviation, and a Kolmogorov–Smirnov test was implemented on each set of measurements to confirm normality. In all cases, our results were normally distributed, with *p* > 0.1 in all cases, and *p* > 0.5 in most cases, with *p* < 0.05 indicating that the data is not normally distributed. Statistical significance was determined using a two-tailed unequal variance Student’s *t*-test.

**Table 1:** Biophysical properties of cells across three exposure conditions.

Parameter (units)	Control	With tubulin disrupter	Hypoosmotic shock
Diameter (µm)	12.9 ± 1.8	13.5 ± 1.7	14.5 ± 1.7*
Refractive index	1.371 ± 0.005	1.367 ± 0.007*	1.361 ± 0.007**
Dry mass (pg)	225 ± 89	228 ± 90	221 ± 55
Fluid volume (µm^3^)	1011 ± 421	1182 ± 464	1502 ± 594*
Dry volume (µm^3^)	164 ± 65	166 ± 66	162 ± 40
Cell volume (µm^3^)	1175 ± 481	1349 ± 518	1664 ± 622*
Cell density (g/mL)	1.053 ± 0.006	1.047 ± 0.010*	1.038 ± 0.009**
Percent water by mass (%)	81.5 ± 2.1	83.4 ± 3.2*	86.4 ± 3.2**
Percent water by volume (%)	85.8 ± 1.7	87.3 ± 2.6*	89.7 ± 2.5**
Brillouin shift (GHz)	7.865 ± 0.049	7.904 ± 0.084	7.687 ± 0.145**
Brillouin linewidth (GHz)	1.279 ± 0.072	1.281 ± 0.085	1.038 ± 0.161**
Longitudinal modulus (GPa)	2.452 ± 0.030	2.479 ± 0.051*	2.345 ± 0.087**
Longitudinal viscosity (Pa s)	0.008 ± 0.0004	0.008 ± 0.0005	0.007 ± 0.001**
Loss modulus (GPa)	0.40 ± 0.02	0.40 ± 0.03	0.32 ± 0.05**
Loss tangent	0.16 ± 0.01	0.16 ± 0.01	0.13 ± 0.02**

*p < 0.05 vs control, **p < 10^–5^ vs control.

Within the control population, the cellular diameter was 12.9 ± 1.8 µm, as determined by the calibrated Hough transform. Upon exposure to tubulin disrupting nocodazole, the mean cellular diameter swelled slightly to 13.5 ± 1.7 µm, though this change was not statistically significant. The cell population under hypoosmotic shock exhibited significant swelling (*p* < 0.05) with a population diameter of 14.5 ± 1.7 µm. These diameters yielded cellular volumes of 1175 ± 481 µm^3^, 1349 ± 518 µm^3^, and 1664 ± 622 µm^3^ for the control, nocodazole, and hypoosmotic conditions, respectively. Importantly, the dry mass of the three cell populations were statistically identical (225 ± 89 pg, 228 ± 90 pg, and 221 ± 55 pg), indicating that an equal amount of non-aqueous material was present in each population per cell, and suggesting that the populations were nearly identical absent the chemical and osmotic insults.

The most obvious changes across the cell populations relate to density, refractive index, and water content. The control population’s refractive index of 1.371 ± 0.005 was reduced slightly to 1.367 ± 0.007 (*p* ~ 0.03) upon tubulin disruption, possibly due to swelling and subsequent water uptake upon disturbance of the microtubule network. The change was even more substantial in the hypoosmotic population, with a refractive index of 1.361 ± 0.007 (*p* < 10^–6^), indicating a substantial drop in the optical density of the cell. These figures are mirrored in the mass density, with densities of 1.053 ± 0.006 g/mL, 1.047 ± 0.010 g/mL (*p* < 0.03), and 1.038 ± 0.009 g/mL (*p* < 10^–6^) for the three respective exposure conditions.

As we measure progressively lower refractive index and density for the exposure populations, we would naturally expect increased water content within the cell. As expected, the mass fraction of water was 81.5 ± 2.1% for the control population, and 83.4 ± 3.2% (*p* = 0.025) and 86.4 ± 3.2% (*p* < 10^–6^) for the nocodazole and hypoosmotic populations, respectively. Similar (though slightly larger) volume fractions for water were observed as expected, caused by the somewhat lesser mass density of water compared with proteins. Nearly identical comparative statistics are observed for the water fractions by volume as by mass, validating our methods.

The Brillouin data indicates similar trends. The hypoosmotic insult substantially reduced the Brillouin shift and Brillouin linewidth, from 7.865 ± 0.049 to 7.687 ± 0.145 GHz (*p* < 10^–5^) and 1.279 ± 0.072 to 1.038 ± 0.161 GHz (*p* < 10^–7^), respectively. Interestingly, the tubulin disrupting agent did not significantly affect either parameter, and the mean of both the Brillouin shift and linewidth were slightly higher than the control population. This is consistent with previous results^53^ which found that in contrast with actin depolymerization, tubulin disruption with nocodazole may actually increase the cellular elasticity.

As stated in the introduction, our system provides the ability to calculate viscoelastic parameters such as the longitudinal modulus, loss modulus, and longitudinal viscosity of the cell, without assumption of the density and refractive index. Taking into account the RI and density from QPI, we compute a longitudinal modulus ($M{^{{\prime}}}$) of 2.452 ± 0.030 GPa for the control, 2.479 ± 0.051 GPa (*p* < 0.05) for nocodazole-treated cells, and 2.345 ± 0.087 GPa (*p* < 10^–5^) for cells under osmotic shock. Unlike the Brillouin shift, where treatment with nocodazole did not significantly increase the Brillouin shift, we observe a minor but statistically significant increase in the longitudinal modulus, indicating that a simple measurement of the Brillouin shift (without incorporating density or RI information) may miss some subtle cyto-mechanical changes.

While effectively unchanged for nocodazole-treated cells, the longitudinal viscosity was substantially reduced for cells under osmotic shock, from 0.008 ± 0.0004 Pa s to 0.007 ± 0.001 (*p* < 10^–7^). The same pattern was observed for the loss modulus and loss tangent, which were reduced from 0.40 ± 0.02 to 0.32 ± 0.05 GPa (*p* < 10^–7^) and 0.16 ± 0.01 to 0.13 ± 0.02 (*p* < 10^–7^), respectively. These results collectively underscore a reduction in viscous-like effects in cells under hypoosmotic shock, which may be partially attributable to increased water content and cytoskeletal damage during colloid-osmotic swelling.

Collectively, the parameters computed by our system broadly characterize the cellular size, mass, density, refractive index, and water content, along with several viscoelastic properties. Violin plots displaying raw datapoints with kernel density estimations for the 15 parameters and three exposure conditions reported in Table 1 are shown in Fig. 3.

A primary benefit of our system is the ability to compare cell populations across multiple parameter dimensions simultaneously. This allows us to check for relationships between parameters which may offer clues to mechanisms of biophysical interaction. Several representative plots demonstrating two-dimensional correlations are shown in Figs. 4 and 5.

We briefly highlight a few interesting relationships. As shown in Fig. 4, we observe a positive relationship between the refractive index and the longitudinal modulus, with coefficients of determination (${R}^{2}$) of 0.18, 0.15, and 0.70 for the control, nocodazole, and hypoosmotic conditions, respectively, and an overall ${R}^{2}$ of 0.46 for all cells. We observed a similar relationship between the density and longitudinal modulus, with ${R}^{2}$ values of 0.19, 0.16, and 0.72 for the three respective conditions and 0.50 overall. The similarity of these trends is primarily due to the close relationship between the refractive index and density themselves, with ${R}^{2}$ > 0.96 for each exposure condition, and ${R}^{2}$ = 0.987 with all cells included. This result makes intuitive sense, as the classic result from Davies and Barer demonstrates that refractive index and concentration of intracellular solutes may be closely approximated with a linear model, independent of solute composition^54,55^.

A significant source of disagreement in the Brillouin literature has regarded the impact of water content on the Brillouin shift, with significant data both supporting^33^ and opposing^34^ the contention that the Brillouin shift is primarily governed by the proportion of the cell constituted by water. Our system resolves this question by measuring the water content (by mass and volume) and Brillouin shift simultaneously. Comparing the water content (by mass) within cells to the Brillouin shift, we observe an overall negative trend between water content and the Brillouin shift, with a coefficient of determination of 0.53. This matches the expected directionality^33^ and would indicate that roughly half of the variance in the Brillouin shift is caused by a change in intracellular water content, while the other half is attributable to other factors. A similar coefficient (${R}^{2}$ = 0.50) was obtained when comparing the mass fraction of water and the longitudinal modulus.

Finally, we compared the Brillouin shift and the longitudinal modulus $({M}^{^{\prime}})$ which was computed using the density and refractive index from QPI. This is a critical comparison, as most studies simply report the Brillouin shift as a surrogate for the mechanical properties of the sample. We are pleased to report an excellent coefficient of determination of 0.998 across all cells measured in the study, with coefficients of 0.989, 0.990, and 0.999 in the sub-populations for the control, tubulin-disrupted, and hypoosmotic conditions, respectively. These results collectively indicate that reporting of the Brillouin shift (rather than the longitudinal modulus) is generally acceptable in scenarios where the mechanical properties of the sample are not required to a very high degree of precision. In methods where a high degree of precision is required, methods such as ours may become necessary to accurately measure more subtle cytomechanical changes independent of RI and density considerations.

Figure 5 displays two-dimensional parameter correlations as in Fig. 4, but using primarily viscous ($\eta$, ${M}^{{^{{\prime\prime}}}}, {\Gamma }_{B}$) rather than elastic (${M}^{^{\prime}}, {\nu }_{B}$) parameters. We briefly highlight a few relationships here. In general, we see a positive relationship between the refractive index and viscous parameters. For example, we find coefficients of determination between the RI and loss modulus ($M{^{{\prime\prime}}}$) of 0.16, 0.08, 0.54, and 0.43 for each of the three exposure conditions and for all cells, respectively. Similar relationships are observed for the cell density versus the loss modulus, and for the cell density versus the longitudinal viscosity. This is expected, given the high degree of correlation between the density and refractive index reported in Fig. 4, and the high degree of correlation (${R}^{2}>0.99$) between the loss modulus and longitudinal viscosity, as reported in Fig. 5. In general, we also observe a negative relationship between the intracellular water content and viscous parameters, with an overall coefficient of determination of 0.44 between the water content (by mass) and the longitudinal viscosity, and 0.46 between the water content and the loss modulus. We also observe positive correlations between the individual viscoelastic parameters, with ${R}^{2}$ of 0.78 for the relationship between the loss modulus and longitudinal modulus, 0.73 for the longitudinal viscosity versus the longitudinal modulus, and 0.996 for the loss modulus versus the Brillouin linewidth, respectively. Similar to our previous argument regarding the Brillouin shift and longitudinal modulus, this last measurement is important, as it ensures that the Brillouin linewidth can be an accurate surrogate measurement for the loss modulus in situations where a high degree of precision is not required.

## Discussion

In this work, we have presented a snapshot colocalized QPI-Brillouin microscope, along with novel processing methods capable of providing up to 15 biophysical parameters from a single simultaneous acquisition. This eliminates the requirement of Brillouin spectroscopy to assume the refractive index and density of cells, while providing several additional parameters of interest, including the intracellular water content by mass and volume, the dry mass, refractive index, density, and numerous biomechanical properties.

Though our measurements in this work have been limited to a single cell type across three exposure conditions, the computed parameters match expected values found elsewhere in the literature. For example, the mean cellular refractive index in the control population was 1.371 ± 0.005, closely matching other reported values from tomographic phase microscopy^56^. The mean cellular density was 1.053 ± 0.006 g/mL, also closely matching literature values^57^. Our longitudinal modulus was either very close to^53^ or slightly lower^27^ than previously reported values in other cell lines, depending on their assumptions of the cellular density and refractive index. We should note here that this parameter is likely to vary depending on the cell line under analysis, and also on ambient conditions such as temperature^58^. The water content measured by our system also matches expected values—though more difficult to measure, it is generally accepted that the water content within cells will be approximately 80–85% of the cell by volume^59^. Our ratio of 85.8 ± 1.7% is reasonable given this condition. While reported measurements of the loss modulus, longitudinal viscosity, and loss tangent are more difficult to find in the literature, our values are close to previously reported values. For example, Liu et al. reported intra-nuclear loss moduli of 0.3–1.5 GPa in osteosarcoma cells^50^, matching the ~ 0.4 GPa reported here. Our measurements of longitudinal viscosity (~ 0.008 Pa s) are quite similar to those reported by Mattana et al.^60^, who measured a value of ~ 6 to 12 centipoise in a control population of NIH/3T3 cells, a value equal to 0.006–0.012 Pa s. Finally, our loss tangents are comparable, but slightly higher than those reported by Chan et al.^48^ in intact mouse ovaries, though this may be explained by a difference in tissue type. Future work will perform these measurements across multiple cell types, and in the presence of additional chemicals and agents, to determine the natural variance of the parameters computable by our system.

The system discussed here used a high numerical aperture objective lens, in which the full spatial bandwidth is utilized for high-resolution QPI imaging, but the back aperture is substantially underfilled by the Brillouin beam, minimizing spectral broadening in Brillouin imaging and allowing for the Brillouin focal volume to sample more of each cell. Even with substantial underfilling of the back aperture, we estimate an effective numerical aperture for the Brillouin beam of ~ 0.9 using a simple geometric model. This equates to an ideal, diffraction-limited beam diameter of ~ 0.72 μm ($d=1.22\frac{\lambda }{NA}$), and an axial spot size ranging from 3 to 6  μm, depending on certain assumptions of the beam quality. In practice, the beam diameter will be slightly larger than this due to the slight but multiplicative effects of aberrations, tolerancing, and heterogeneities in the sample. Given that the minimum diameter of our cells is ~ 10  μm, we felt that this beam was well-tuned for sampling as much of the cellular volume as possible under the natural constraint of an anisotropic point-spread function. Our attempts to further underfill the objective’s aperture (and thus, sample more of the cellular volume) resulted in increased elastic scattering and poor Brillouin signal quality, likely resulting from increased scattering at the cell-glass interface from a lengthened focal zone. Future work may utilize antireflection-coated coverglass to allow further optimization of the Brillouin focal volume.

In some experiments, a low coefficient of determination (${R}^{2}<0.3$) was reported for certain exposure conditions, whereas a high correlation was observed for the overall population, encompassing all exposure conditions. In many cases, this may be affected by lesser natural variance for cells within a given population, artificially increasing the effects of measurement uncertainty. For example, we observed a significant relationship between the cell density and longitudinal modulus overall (${R}^{2}$ = 0.5), though there was little relationship for the nocodazole-treated sub-population (${R}^{2}$ = 0.16) and a strong dependence in hypoosmotic cells (${R}^{2}$ = 0.72). However, a wider range of values was reported for both the density and longitudinal modulus in hypoosmotic cells, potentially reducing the impact of a fixed measurement uncertainty in this more heterogenous sub-population. In such instances, the overall correlation (across all exposure conditions) is useful for determining the relationship between biophysical parameters.

Prior to computing the average RI of each cell, our system computes a spatial refractive index map $\Delta n\left(x,y\right)$, which could prove useful in some applications, as different subcellular compartments are known to exhibit heterogenous refractive indices^41^. In this work, we chose to report the average cellular refractive index only, for several reasons. First, QPI alone is not capable of distinguishing between intracellular compartments, limiting the usefulness of this information. Second, at each location within $\Delta n\left(x,y\right)$, the refractive index has already been spatially integrated along the optical axis, and presumably, will contain information from different subcellular compartments aligned along this dimension. Most importantly, care was taken to match the point spread function of the Brillouin beam as closely as possible to the entire cell, consistent with our goal of building a device which enables rapid measurements of whole-cell biophysical parameters. Since the Brillouin-derived measurements require a refractive index from the same spatial location as the Brillouin beam, a whole-cell RI is most useful here. Still, the spatial refractive index map is available to users of our system, and may be combined with an additional modality in the future (for example, correlative fluorescence imaging^61^ or two-dimensional Brillouin mapping^28^) to study the heterogeneity across subcellular compartments.

In Brillouin imaging, care must be taken to ensure that the Brillouin beam is not phototoxic to the sample. It has previously been reported that damage to cells can occur in Brillouin imaging when (on average) ~ 3 J of 532 nm light have been delivered to the sample^62^. At our power of ~ 3.8 mW, this will occur after slightly more than 13 min of continuous exposure, or around 400 times longer than the two-second exposure used in this work. Additionally, that study was performed with a similar numerical aperture (0.6 NA versus ~ 0.9 effective NA in our work) alleviating concerns about matching irradiance at the cell, especially given our large safety factor of ~ 400. Future work may increase the Brillouin beam intensity to enable shorter exposure times, and ultimately, faster throughput.

In any cytometric application, the throughput (in cells analyzed per minute) is a consequential factor in determining the usefulness of the method. In this work, we pursued a proof-of-concept design with manual targeting of individual cells, permitting the analysis of ~ 3 to 5 cells per minute by a skilled operator. Because manual targeting was the primary bottleneck to throughput, we utilized a somewhat long Brillouin integration time of 2 s to improve SNR. Of course, microfluidic architectures exist which could rapidly funnel cells through the QPI and Brillouin focal volumes. Under this paradigm, the throughput would be theoretically limited by the Brillouin integration time, the state-of-the-art being somewhat more restrictive than QPI^63^ due to the lower levels of photon efficiency in Brillouin scattering. Under such a configuration, the minimum time required to analyze one cell would be on the order of tens of milliseconds^64^, permitting a theoretical upper limit of ~ 25 cells per second, assuming negligible transition time between cells. Further considerations, such as detector noise, power at the sample, and post-processing times may further constrain the throughput. Future work will optimize the system presented here for high-throughput biophysical cytometry of large cell populations.

Attentive reading of our methodology will reveal that the refractive index of the cell was computed at the wavelength of the QPI source (${\lambda }_{Q}$ = 633 nm) while computing the Brillouin shift requires the refractive index at the wavelength of the beam used for Brillouin scattering (${\lambda }_{B}$ = 532 nm). While chromatic dispersion is non-zero, literature values show this to be < 10^–5^ nm^−1^^23^, suggesting that this issue will affect the refractive index by < 0.001. Future systems may choose a suitable filtering scheme to more closely align the QPI and Brillouin wavelengths, effectively eliminating this source of error.

The spherical geometry assumption used in this work is clearly a powerful one, as it allows decoupling of the RI-thickness ambiguity which is common in QPI. While powerful, this approach includes obvious drawbacks. Studying time-dependent effects on a single cell is limited to durations shorter than the time required for cells to adhere to the surface of the imaging dish and lose their spherical geometry, which is typically on the order of 2–4 h. This issue can be mitigated using suspension cell lines such as Jurkat or U937, which do not adhere, and maintain a roughly spherical geometry naturally. Temporary adhesion may be used to adhere these cells to the dish^24^, preventing escape from the Brillouin beam during long exposures. Alternative methods to decouple RI and sample thickness, such a tomography^29^ or medium substitution^65^ are capable of isolating the refractive index, but defeat the single-shot advantage of the system employed here. “Transmission-through-dye” approaches may be used to compute the volume of non-spherical cells using the absorption of an exogenous dye as an indicator of cell height^66,67^, though this method requires an external contrast agent. Finally, the spherical cell assumption may prove useful in flow cytometric applications, where cells are naturally more round, and Brillouin spectroscopy systems have already been developed^68^.

## Conclusion

In this work, we have introduced a dual-modality image cytometry system based on quantitative phase imaging and Brillouin spectroscopy which can acquire more than a dozen biophysical properties of a single cell simultaneously. Using a geometric simplification consistent with the behavior of freshly plated cells, we use complementary information from our two modalities to comprehensively characterize the refractive index, mass density, water content, and viscoelasticity of cells, among other properties. Our system was validated across three distinct cell populations. We look forward to utilizing this system for a broad array of biophysical applications.

## Data Availability

Data underlying the results presented in this paper may be obtained from Zach Steelman (zachary.steelman.1@us.af.mil) upon reasonable request.
